# New-Onset Left Bundle Branch Block After TAVI: An Updated Review

**DOI:** 10.3390/jcm15083016

**Published:** 2026-04-15

**Authors:** Juan Ignacio Mayol, Guillem Muntané-Carol, Montserrat Gracida, Andrea Ruberti, Ana Marcano, Gerard Roura, Neus Salvatella, Luis Teruel, Lara Fuentes, Josep Gómez-Lara, Rafael Romaguera, Josep Comín-Colet, Joan Antoni Gómez-Hospital

**Affiliations:** 1 Centro Cardiológico Americano, Sanatorio Americano, 11300 Montevideo, Uruguay; juanimayol8@hotmail.com; 2 Cardiology Department, Bellvitge University Hospital, 08907 L’Hospitalet de Llobregat, Catalunya, Spain; 3 Bio-Heart Cardiovascular, Respiratory and Systemic Diseases and Cellular Aging Program, Bellvitge Biomedical Research Institute (IDIBELL), 08907 L’Hospitalet de Llobregat, Catalunya, Spain

**Keywords:** transcatheter aortic valve implantation, conduction disturbances, aortic stenosis, new-onset left bundle branch block

## Abstract

Transcatheter aortic valve implantation (TAVI) has become the preferred treatment for patients with symptomatic severe aortic valve stenosis. Newer-generation devices, increased operator experience, and improved patient selection have contributed to a reduction in complication rates. However, the occurrence of new-onset left bundle branch block (LBBB) after TAVI remains high, and currently it is the most common complication associated with the procedure. This review discusses the current understanding of new-onset LBBB, including its causes, incidence, clinical outcomes, and management strategies.

## 1. Introduction

Degenerative aortic stenosis (AS) is a major health concern and the most common primary valve disease requiring intervention in Europe and North America. Its prevalence is projected to rise significantly as the population ages [1]. Currently, no medical treatment has been shown to improve survival in patients with severe AS [2].

The prognosis becomes dismal once symptoms manifest, with five-year survival rates ranging from 15 to 50% [3]. According to recent guidelines, early intervention should be considered even in asymptomatic patients, regardless of the presence of additional adverse prognostic features [4,5].

While surgical aortic valve replacement (SAVR) has traditionally been the gold standard, transcatheter aortic valve implantation (TAVI) has become a well-established treatment. In recent years, TAVI has gained prominence as the preferred option for older patients, regardless of their surgical risk [4,5,6,7,8,9,10,11,12].

Fortunately, periprocedural complications have decreased as experience and technology evolved. However, new-onset conduction disturbances (CDs), specifically left bundle branch block (LBBB), remain among the most frequent complications after TAVI [13]. Although the impact of LBBB on heart failure hospitalization, cardiovascular mortality, and all-cause mortality has been debated [14,15], its detrimental effects on left ventricular synchrony and the increased risk of permanent pacemaker implantation (PPI) may negatively influence clinical outcomes [16,17].

Furthermore, a significant lack of consensus persists regarding the definition and management of this condition across the TAVI field. This has led to substantial variability in clinical practice, ranging from conservative observation to prophylactic PPI [18,19]. This review aims to provide an updated overview of persistent new-onset LBBB, focusing on its incidence, clinical impact, and management strategies.

## 2. Methodology

This manuscript was conducted as a narrative review. A structured literature search was performed using major electronic databases, including PubMed, Embase, Cochrane, Scopus, and Web of Science. The search covered studies published from 2010 to 2025 using the terms “TAVI” or “TAVR” AND “left bundle branch block,” combined with “conduction disturbances”, “clinical outcomes”, “pacemaker implantation”, “heart failure”, and “management”. In addition, the reference lists of selected articles and relevant review papers were manually screened to identify additional pertinent studies. Original studies, randomized trials, registry analyses, and meta-analyses reporting data on incidence, predictors, pathophysiology, clinical impact, or management of new-onset LBBB after TAVI were included. Given the narrative design of the review, no formal systematic quality scoring tool was applied. The available evidence was synthesized descriptively and organized into thematic sections to provide a comprehensive and clinically oriented update.

## 3. New-Onset LBBB: Timing and Incidence

The reported incidence of new-onset LBBB after TAVI varies widely, ranging from 13.3 to 39% [20,21]. This broad range is primarily attributed to variations in transcatheter heart valve (THV) designs, baseline population characteristics, and inconsistencies in defining new-onset LBBB based on electrocardiogram (ECG) acquisition time points. Notably, despite advancements in operator experience and newer-generation valve systems, the incidence has not decreased significantly over the years [21]. In this regard, recent large-scale evidence from the STS/ACC TVT Registry (2016–2022) involving over 200,000 patients reported an overall new-onset LBBB incidence of 16.3%. Interestingly, this work revealed only a modest decline in LBBB rates over the study period—from 19.9% in 2016 to 14.4% in 2022. Despite this reduction, the incidence remains notably high, underscoring that new-onset LBBB is far from being a resolved issue in the TAVI setting [22].

While approximately 90% of new-onset LBBB cases occur within the periprocedural period (the first 24 h), roughly half of these persist beyond hospital discharge [23]. Following hospitalization, previous data have shown diverging clinical outcomes in these patients, with rates of PPI and spontaneous resolution of the LBBB at one year of 10% and 33%, respectively [24,25].

Among first-generation THV systems, the incidence of LBBB was markedly higher with the self-expandable CoreValve system compared to the balloon-expandable Edwards SAPIEN/SAPIEN XT valve (18–65% vs. 4–30%, respectively) [13,26]. These two main valve types—self-expanding and balloon-expanding—differ in design and deployment mechanisms. Self-expanding valves, made with nitinol-based stent frames, gradually expand upon exposure to body temperature, continuing to open for several minutes after deployment until reaching their final shape and size [27]. This prolonged expansion may contribute to differences in LBBB frequency between valve types, though this relationship has not been demonstrated in the latest available randomized data [28]. Between self-expanding valves, Pellegrini et al. demonstrated in a SCOPE2 sub-analysis that the ACURATE Neo (Boston Scientific) showed lower rates of LBBB compared to the CoreValve Evolut device (Medtronic) [29].

**Figure 1:**
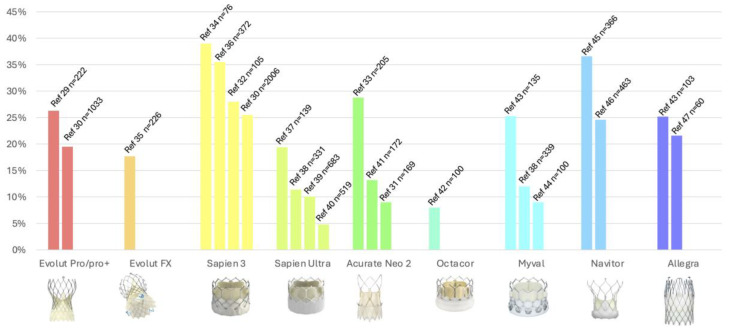
New-onset left bundle branch block (LBBB) rates using newer-generation transcatheter heart valves [29,30,31,32,33,34,35,36,37,38,39,40,41,42,43,44,45,46,47].

On the other hand, the reported incidence of new-onset LBBB with newer-generation valves remains limited. Figure 1 shows data in current newer THV systems, ranging from 9.0% to 39% [29,30,31,32,33,34,35,36,37,38,39,40,41,42,43,44,45,46,47]. Of note, recent data using a propensity match cohort showed that the use of ACURATE neo2 (now discontinued from the market) appears to be linked to lower rates of new-onset LBBB compared to both the Edwards S3 and Evolut Pro [31].

## 4. Tavi and Conduction System

**Figure 2:**
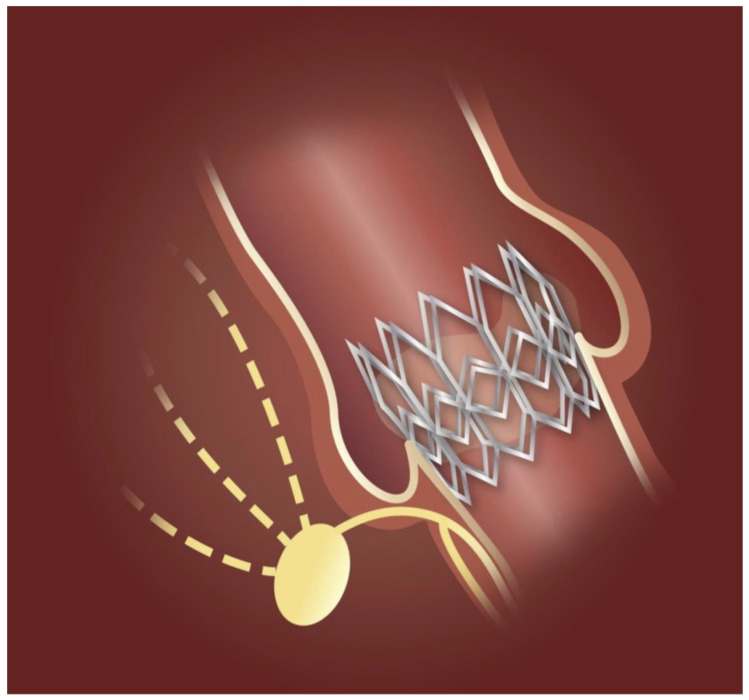
Schematic illustration of the anatomical relationship between the transcatheter heart valve and the cardiac conduction system. Reproduced with permission from the authors (Muntané-Carol, G., et al. [21]).

**Figure 3:**
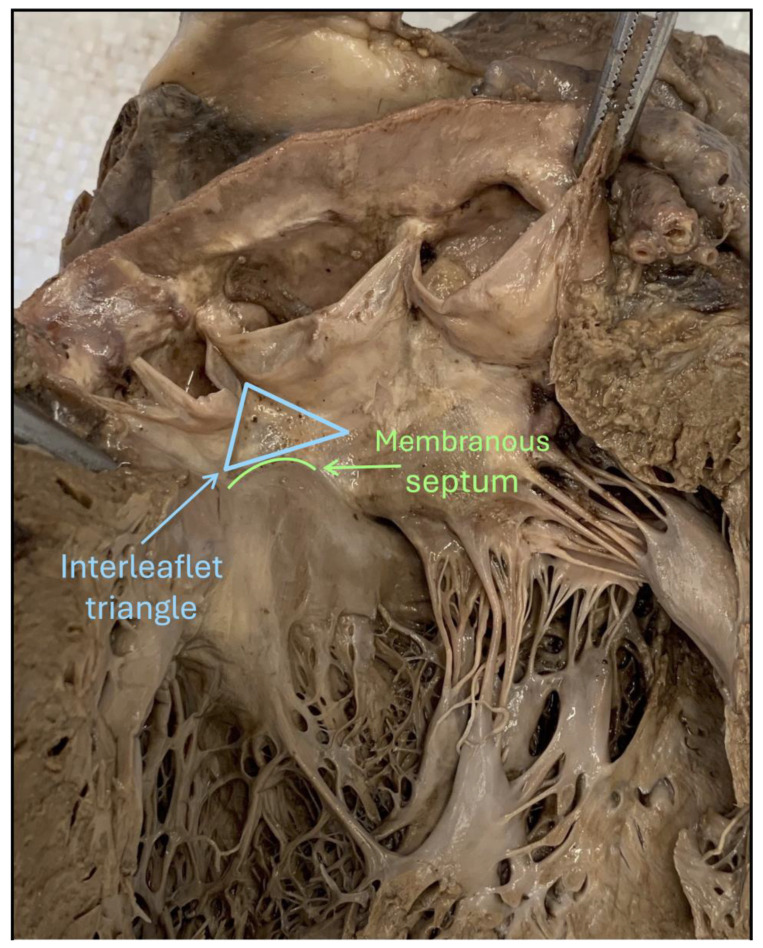
The opened aortic root from the left ventricle, showing the most superior part of the left bundle branch as it originates from the branching component of the conduction axis (interleaflet triangle and membranous septum). Courtesy of Dra. Garretano, Alejandra.

The development of peri-procedural CDs during TAVI is primarily caused by direct mechanical trauma to the conduction system, specifically the bundle of His and the left bundle branch (Figure 2). These structures are anatomically situated near the base of the right and non-coronary leaflets [21]. In this region, the conduction system penetrates the membranous septum (MS), making it highly vulnerable to compression by the aortic valve prosthesis (Figure 3). The MS is divided into two portions: the supra-annular and infra-annular [48]. Precise measurement of the infra-annular portion during pre-procedural planning is crucial, as a shorter infra-annular length strongly correlates with an increased risk of CDs [49,50]. Data from the INTERSECT registry identified MS length as an independent predictor of PPI [51]. Three risk categories for CDs were identified based on MS length: less than 3 mm (high risk), between 3 mm and 7 mm (intermediate risk), and greater than 7 mm (low risk) [51].

Higher implantation of the prosthetic valve has been linked to a lower incidence of new-onset LBBB post-TAVI [21]. This can be attributed to a reduced contact surface with the MS, thereby decreasing mechanical stress [52,53]. A recently published technique involves the use of intracardiac echocardiography to measure the length of the MS and guide final prosthetic valve positioning, resulting in a substantial reduction in CDs, including new-onset LBBB [54,55].

On the other hand, the use of low radial force valves was associated with a diminished inflammatory response in one study and lower occurrences of new-onset LBBB compared to high radial force valves [56]. Furthermore, prosthetic overexpansion of the native annulus (prosthesis oversizing) has been identified as one of the main predictors of new-onset LBBB post TAVI [57,58].

CDs can occur at various stages of the procedure, as mechanical interaction can happen during pre- or post-dilatation and even during wire placement within the left ventricle [21]. In this line, previous data showed that approximately 50% of CDs during the TAVI procedure may occur before valve implantation [59]. Therefore, minimizing manipulation in the left ventricular outflow tract could help to reduce damage to the conduction system. A multicenter registry reported a low incidence of new-onset LBBB of 10.3%, with only 2.3% requiring PPI [60]. To minimize trauma to the aortic annulus and left ventricular outflow tract, pre-dilatation was performed in all patients using a balloon that was 1–3 mm smaller than the perimeter-derived annular diameter. If post-dilatation was necessary, a balloon that was 1.2 ± 0.9 mm smaller than the perimeter-derived diameter was used [60].

Finally, several patient-specific factors were associated with new-onset LBBB following TAVI. Specifically, baseline first-degree atrioventricular block and a wider pre-procedural QRS duration have been shown to increase the likelihood of new-onset LBBB [52]. Additionally, the presence of severe annular calcification is significantly associated with a higher incidence of post-procedural LBBB [57]. A study utilizing 3D-printed silicone annular models demonstrated that elevated calcification in the left coronary cusp (exceeding 209 mm^3^) can cause an off-center shift of both the valvuloplasty balloon and the THV toward the right and non-coronary cusp commissure [61]. This displacement results in direct contact and potential mechanical trauma to the conduction tissue, specifically the left bundle branch. Interestingly, while these patients exhibited higher rates of PPI, they did not show a corresponding increase in new-onset LBBB [61]. Furthermore, low coronary heights (under 11 mm) have been identified as a significant risk factor for LBBB, as operators often opt for a deeper valve implantation to mitigate the risk of coronary obstruction [57]. Finally, observational data indicate that patients with bicuspid aortic valves are particularly predisposed to this complication, as they tend to present with significantly shorter MS lengths, which inherently increase the risk of CDs [62].

## 5. Clinical Impact

The clinical impact of new-onset LBBB has not been well established due to heterogeneous evidence across studies, often influenced by differences in follow–up duration, baseline population characteristics, and varying definitions of the conduction defect itself [63]. Traditionally, research in this field has focused on three primary concerns: progression to high-grade atrioventricular block (HAVB) requiring PPI, the development or worsening of heart failure, and increased long-term mortality.

### 5.1. Permanent Pacemaker Implantation (PPI)

The incidence of PPI after TAVI is significantly higher than after SAVR. PPI following the procedure ranges from 3.4% to 51%, depending on the study, compared to approximately 5.5% for SAVR [20]. Several key factors predict PPI after TAVI, including right bundle branch block, first-degree atrioventricular block, self-expandable valves, implantation depth, and MS length [13].

New-onset LBBB has been consistently identified as a potent predictor of subsequent PPI. Data from the PARTNER II trial showed that new-onset LBBB doubled the PPI rate after TAVI, a finding supported by a previous meta-analysis [17,64]. Additionally, a systematic review of over 29,000 patients confirmed that new-onset LBBB independently predicted PPI in intermediate-risk patients [65]. Two subsequent meta-analyses further demonstrated a significant association between LBBB and PPI at 30 days, one year, and two years [66,67]. However, the use of routine prophylactic PPI is not indicated in this population. This conservative approach is driven by the high degree of data heterogeneity and the significant rates of LBBB regression observed during follow-up, which warrant a strategy of clinical and electrocardiographic surveillance rather than prophylactic PPI [68,69,70].

### 5.2. Heart Failure and Mortality

LBBB has traditionally been associated with an increased risk of cardiovascular morbidity and mortality [71,72]. It impairs both systolic and diastolic LV function through intraventricular desynchrony, contributing to a cycle of LV wall stress, asymmetric hypertrophy, hypoperfusion, and dilatation that progressively worsens LV function [72,73]. The Framingham study showed that 28% of asymptomatic LBBB patients developed HF with a median of 3.3 years after the LBBB pattern recognition, which is seven times higher than the population without LBBB in the same period [74]. Most patients with LBBB have underlying structural heart disease, such as coronary artery disease or a form of cardiomyopathy. Consequently, isolated LBBB without heart disease is rare [75,76]. In a large cohort of heart failure patients, LBBB was present in 25% of cases and was independently associated with a significantly higher risk of 1-year mortality, serving as a strong prognostic marker regardless of other risk factors [77]. Despite these associations, it remains unclear whether LBBB serves as a predictor, cause, or consequence of myocardial dysfunction [73].

Several studies highlight the association between persistent LBBB after TAVI and heart failure. New-onset persistent LBBB post-TAVI has been linked to impaired reverse cardiac remodeling, a decline in left ventricular ejection fraction (LVEF) at one year, and increased rates of heart failure hospitalization, cardiovascular mortality, and all-cause mortality [78,79,80,81]. This negative impact on left ventricular systolic function is particularly pronounced in patients with baseline LVEF < 50%, as their LVEF fails to recover, potentially worsening their prognosis [80].

The association between new-onset LBBB and hard clinical endpoints such as hospitalization for heart failure and mortality has historically been debated due to conflicting observational data [17,66,82,83,84,85]. However, the previously cited STS/ACC TVT Registry confirmed that new-onset LBBB is an independent predictor of adverse outcomes at one year, with significantly higher rates of all-cause mortality (12.3% vs. 10.1%; HR 1.19) and heart failure hospitalizations (8.1% vs. 6.3%; HR 1.25) [22]. These findings corroborate previous meta-analyses and address the discrepancies found in earlier, smaller observational studies that may have been underpowered to detect a significant association [17,66,82,83,84,85].

In summary, current evidence indicates that new-onset LBBB is associated with impaired myocardial recovery, a higher burden of heart failure hospitalizations, and poorer long-term survival following TAVI.

## 6. Management and Future Perspectives

The management of new-onset LBBB patients remains an unmet need, and its approach after the procedure has been largely debated since the beginning of TAVI. In recent years, various strategies have been implemented [18,19]. These include clinical observation, prophylactic PPI [86], extended ambulatory ECG (AECG) monitoring [69] or PPI based on an electrophysiology study (EPS) result [68,87]. Nowadays, there is a growing shift toward a more tailored management that aims to balance the risks of delayed arrhythmic events with the potential complications of unnecessary PPI.

Given the risk of progression to HAVB, the 2018 AHA/ACC/HRS Guidelines recommend careful clinical and electrocardiographic monitoring (Class IIa), while assigning a Class IIb recommendation for PPI [86]. In contrast, the 2021 European Society of Cardiology (ESC) Guidelines, along with a previous expert consensus published by Rodés-Cabau, provide more specific criteria for management [68,69]. In patients with new-onset LBBB and significant conduction delay—defined as a QRS duration >150 ms or a PR interval > 240 ms—it is recommended either an EPS after the third post-procedural day or a period of short-term AECG monitoring [69].

The recently published PROMOTE study (Prospective Validation of a Pre-specified Algorithm for the Management of Conduction Disturbances Following TAVR) has provided significant insights into the natural history and management of new-onset LBBB [88]. This multicenter study, which enrolled 2110 patients following the Rodés-Cabau post-procedural protocol including AECG monitoring, reported that 35.8% (n = 756) of patients developed new-onset LBBB. At 30-day follow-up, the conduction abnormality remained stable in 31.2% of cases, while nearly half (49.4%) exhibited partial or complete resolution. Notably, 17.2% of these patients required PPI, with the vast majority (93.1%) receiving the implant during the index hospitalization (median of 4 days post-TAVI). For the remaining few requiring PPI after discharge, significant bradyarrhythmias were detected by AECG monitoring [88].

Given the high rate of spontaneous LBBB resolution during the initial hospital stay, prolonged temporary pacing is no longer recommended, a shift in strategy proposed by a recent consensus update from the same investigators [89]. However, close clinical surveillance for at least 24–48 h following the procedure is mandatory to detect bradyarrhythmic episodes. In this regard, the close monitoring of the PR interval is key, as its prolongation is associated with HAVB.

### 6.1. Ambulatory ECG Monitoring

AECG monitoring has emerged as a fundamental tool for detecting CDs across various clinical scenarios. Currently, multiple technologies are available, including standard 24–48 h Holter monitors, patch-type devices, external loop recorders, mobile cardiovascular telemetry—which may allow for real-time ECG analysis—and implantable cardiac monitors (ICM) [90].

Some studies have evaluated the clinical utility of these tools in the global post TAVI setting, including patients with new-onset LBBB [91,92,93]. While this data has shown that the incidence of clinical bradyarrhythmias in patients with normal ECG at discharge is very low [94], those with new-onset LBBB remain at greater risk, and AECG monitoring may be considered [69].

The MARE study, which used an ICM in the specific setting of new-onset LBBB, established the safety of AECG monitoring [24]. The study included 103 consecutive patients and showed that up to 16% of patients with persistent LBBB after TAVI suffered from HAVB at a 2-year follow-up, with 50% occurring within the first month [95]. Hence, these results suggest the lack of significant delayed damage to the conduction system and do not support prophylactic PPI in these patients. However, the use of continuous AECG monitoring during the first weeks after the procedure might be evaluated, as recommended in some expert consensus and review articles [69,90]. In the PROMOTE cohort, nearly 10% of PPI in the LBBB group occurred after hospital discharge, triggered by significant arrhythmias detected via AECG. These findings underscore the clinical utility of ambulatory monitoring in patients with persistent LBBB, as it facilitates the detection of late conduction-system events [88].

### 6.2. Electrophysiology Study (EPS)

Given the inherent limitations of the surface electrocardiogram in accurately predicting the clinical progression of patients with new-onset LBBB following TAVI, several studies suggest that the EPS could be useful as a diagnostic tool [63,96,97,98]. By allowing for a direct assessment of the infra-nodal conduction system through the measurement of the His-ventricular (HV) interval, the EPS helps discriminate patients at the highest risk of progressing to advanced atrioventricular block. Specifically, an HV interval ≥ 70 ms post-procedure has been proposed as a predictor for pacing [68]. Conversely, a normal HV interval may identify a low-risk subgroup. However, it is important to underscore that the clinical utility of EPS in the specific context of TAVI is based on the extrapolation of data from symptomatic patients outside the setting of TAVI. Traditionally, HV interval thresholds have been validated in populations with chronic, progressive conduction disease, rather than in the scenario of acute, localized mechanical trauma induced during TAVI. Consequently, despite its use, there is still a lack of prospective evidence validating these parameters in the TAVI setting. In this regard, the recently published LBBB-TAVI study added new data regarding this issue [87]. Masssouillé et al. included 183 TAVI recipients with new-onset LBBB. Patients with an HV interval > 70 ms (n = 47) received a PPI capable of recording CDs, while those with an HV interval < 70 ms (n = 136) underwent 12-month AECG monitoring. Although patients with HV >70 ms had significantly more high-grade AV conduction disorders (53.2% vs. 22.8%; *p* = 0.001), the results also highlighted the limitations of this threshold. Notably, half of the patients who received a PPI did not experience HAVB or complete heart block (CHB) episodes, and one out of five patients with a normal baseline HV interval still developed advanced block [87]. These findings are consistent with a meta-analysis of 18 studies, which found that while over half of the patients who received a PPI based on abnormal EPS findings became pacemaker-dependent during follow-up, a significant proportion could have likely avoided the intervention with better stratification [99]. This underscores that despite the utility of EPS, further research is required to refine its predictive accuracy post-TAVI.

### 6.3. Management Algorithms

**Figure 4:**
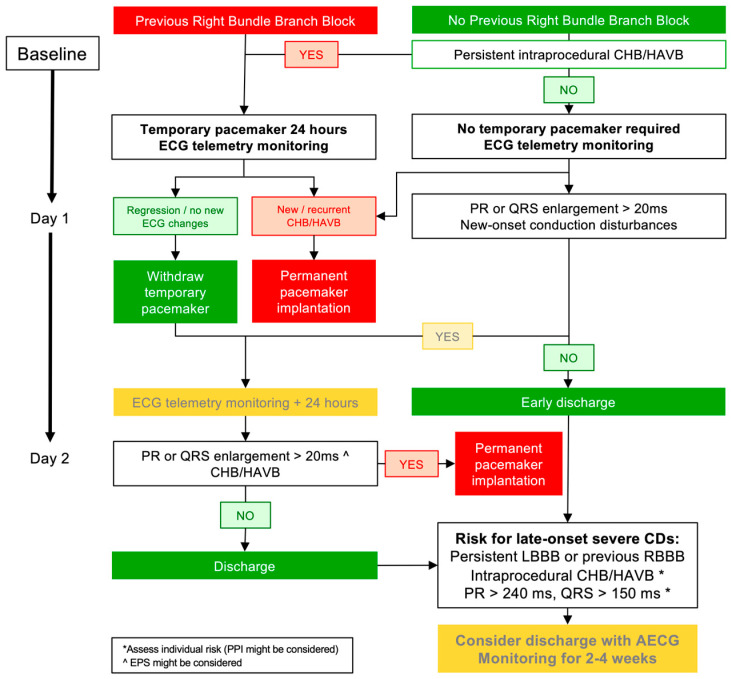
Global management and discharge day proposal according to the occurrence of new-onset CDs. AECG: Ambulatory electrocardiogram; CDs: Conduction disturbances; CHB: Complete heart block; ECG: Electrocardiogram; EPS: Electrophysiological study; HAVB: High-grade atrioventricular block; LBBB: Left bundle branch block; RBBB: Right bundle branch block. Reproduced with permission from the authors (Nuche, et al. [89]).

**Figure 5:**
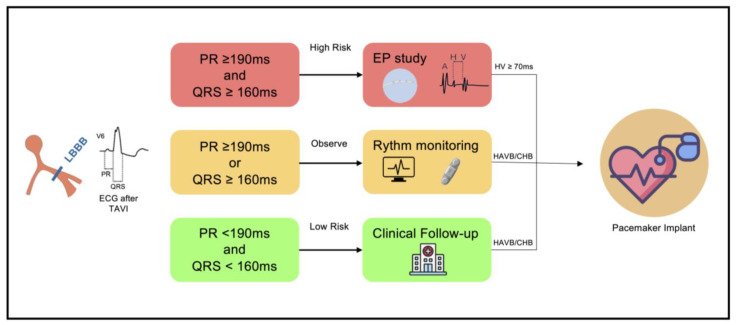
Proposed risk stratification of patients with new-onset LBBB after TAVI based upon the postprocedural ECG. CHB = complete heart block; ECG: Electrocardiogram; EP: electrophysiology; HAVB: high-grade atrioventricular block; adapted with permission from the authors (Badertscher, et al. [100]).

Two algorithms on the management of new-onset CDs post TAVI are illustrated in Figure 4 and Figure 5. It is important to note that these algorithms are based on expert recommendations, and there is currently no randomized prospective evidence demonstrating that they improve hard endpoints. Nuche et al. recently proposed and updated global algorithm based on the presence of intraprocedural heart block and ECG-CDs (Figure 4), which serves as an update from the previously cited consensus [69,89]. Regarding new-onset LBBB, this algorithm avoids the routine use of temporary pacemaker following the procedure. It recommends hospital discharge at day 2 post-TAVI if stable ECG with PR interval and QRS complex of less than 240 ms and 150 ms, respectively (Figure 4) [89]. For the remaining high-risk patients—those exhibiting PR or QRS prolongation > 20 ms, or PR > 240 ms and QRS > 150 ms—the algorithm suggests that PPI, EPS, or discharge with AECG monitoring may be considered according to local protocols.

Figure 5 illustrates a specific algorithm for new-onset LBBB patients proposed by Badertscher et al. [100]. The authors introduce a novel, simplified ECG-based algorithm designed to improve the prediction of infranodal conduction delay (HV interval ≥ 70 ms in EPS) in patients with LBBB after TAVI [100]. By analyzing a multicenter cohort, the authors proposed a risk-stratified approach divided into three categories: (i) Patients with a PR interval ≤ 190 ms and a QRS duration ≤ 160 ms are classified as low risk, allowing for safe hospital discharge without further intervention. (ii) Patients with PR ≥ 190 ms or QRS ≥ 160 ms are classified as intermediate risk, which warrants extended hospitalization and discharge with AECG monitoring. (iii) Patients with PR ≥ 190 ms and QRS ≥ 160 ms are classified as high risk, which showed the strongest correlation with significant conduction disease and mandates an EPS to guide definitive pacing decisions. Of note, previous data showed an increased risk of delayed HAVB and even sudden death in this group of patients [14,94,101]. Although the study does not specify a mandatory discharge timeline, a 2-day hospital stay for the low-risk group seems reasonable, whereas a 3 to 4-day stay is more appropriate for intermediate and high-risk patients to allow for additional monitoring or the completion of invasive studies.

### 6.4. Future Data

The occurrence of TAVI-related CDs may be partly attributed to an acute inflammatory response triggered by the mechanical trauma of the procedure. Consequently, there is growing interest in exploring peri-procedural anti-inflammatory therapies to mitigate the incidence of new-onset CDs. To date, however, retrospective single-center studies have failed to demonstrate a significant reduction in PPI rates associated with glucocorticoid exposure [102,103,104].

Notably, the randomized GLUCO-TAVR (Glucocorticoids in Patients Undergoing TAVR to Prevent Pacemaker Implantation) trial, which included 100 patients, found that methylprednisolone administered one hour before TAVI, followed by five days of prednisolone, did not significantly reduce the rates of PPI or new-onset LBBB, without differences in safety outcomes. However, this was an exploratory pivotal trial with a relatively small sample size, which may have limited its statistical power [105].

In addition, the ongoing EAGLE-TAVR (NCT06762145) trial is currently comparing placebo versus intravenous methylprednisolone for three days, starting on the day of the procedure. The primary endpoint of this study is the incidence of persistent LBBB at 30 days, which will provide further insights into the role of anti-inflammatory therapy in this setting. Finally, the Co-STAR trial (NCT04870424) aimed to evaluate the effect of colchicine on new-onset CDs but was terminated prematurely owing to an unexpected increase in the incidence of stroke among patients receiving colchicine [106].

Furthermore, the transient nature of new-onset LBBB is underscored by the early recovery of ECG-CDs observed after discharge and the frequent absence of significant ventricular pacing beyond the first few weeks post-TAVI [107,108]. This potentially reversible inflammatory nature may entail a risk of overtreatment if PPI is based on an EPS result conducted early following the procedure. The TAVI-REVERSE study (NCT06481137), a prospective multicenter trial, aims to address this by enrolling patients with a clinical indication for an EPS. Notably, patients with an initially positive EPS who receive a PPI will undergo a repeat EPS one month later. This design will allow for a precise assessment of conduction recovery rates and the identification of predictive factors, potentially leading to the proposal of revised thresholds regarding EPS in the post-TAVI setting.

On the other hand, Chang et al. recently explored the use of a temporary–permanent pacemaker, utilizing an active fixation lead connected to an externalized pulse generator—as a 30-day “bridge to decision” for patients with new-onset ECG-CDs [109]. Remarkably, at 30 days, 76% of the patients (including 77% who initially presented with CHB) no longer required pacing, allowing for lead removal without PPI. Although two adverse events related to the lead were reported, there was no mortality [109]. This strategy confirms a high rate of conduction recovery within the first month and significantly reduces unnecessary PPI, though these results require confirmation in larger, randomized cohorts.

Finally, while AECG monitoring has shown promising results for managing new-onset CDs, additional evidence is required to confirm its clinical utility and cost-effectiveness. The risk of overtreatment remains a concern; therefore, randomized studies are essential to evaluate the impact of AECG on hard endpoints such as sudden cardiac death, unplanned hospitalizations, and overall length of stay.

The most relevant upcoming studies focused on new-onset CDs and LBBB management after TAVI are summarized in Table 1.

## 7. Discussion

Despite the previously mentioned recommendations [69,89], several critical gaps persist in the management of persistent new-onset LBBB following TAVI. The optimal duration in-hospital length of stay, the most appropriate timing for PPI, or the definitive role of EPS in guiding clinical decision-making remain subjects of ongoing debate.

The management of these patients is particularly challenging due to the heterogeneous nature of TAVI-induced CDs. While some LBBB cases are transient and resolve spontaneously, others carry a high risk of progressing to delayed HAVB/CHB. To date, no study has identified definitive predictors of LBBB regression [25]. This clinical uncertainty often leads to prophylactic PPI in patients who might not have experienced HAVB/CHB during their hospitalization. In this context, a pre-specified sub-analysis of the PROMOTE study highlighted this trend: 90% of prophylactic PPIs in the cohort were indicated for new-onset persistent LBBB (QRS > 150 ms and/or PR > 240 ms) [110]. However, the median ventricular pacing rate at 30 days was significantly lower in the prophylactic group compared to patients who experienced actual HAVB/CHB events (2% vs. 73%; *p* < 0.001) [110]. These findings underscore a significant risk of overtreatment and emphasize the urgent need for robust predictors of both conduction recovery and progression to high-grade block.

The management of CDs following the TAVI procedure will continue to evolve in the coming years as a key strategy to improve outcomes. Regarding new-onset LBBB, operators may focus on strategies to minimize the potential harm to the conduction system. First, pre-procedural planning should include accurate valve sizing, precise evaluation of MS length, and, when feasible according to local practice, a tailored selection of the THV. Second, intra-procedural optimization aims to reduce interaction with the conduction tissue by avoiding unnecessary pre- and post-dilatation and adopting specific deployment techniques, such as the cusp-overlap view, to achieve higher implantation depths.

Regarding temporary pacing, intraprocedural requirements have decreased with the adoption of left ventricular pacing via the TAVI guidewire [111]. For patients with new-onset LBBB, a temporary pacemaker is only indicated if intraprocedural HAVB/CHB occurs. Nevertheless, maintaining central venous access (preferably jugular) is recommended, as the need for urgent pacing may arise post-procedure. This sheath can typically be removed after 24 h of ECG stability.

In summary, for those patients with a stable ECG or regression of the LBBB, PR interval < 240 ms, and QRS < 150 ms, discharge 48 h after TAVI appears safe (Figure 4 and Figure 5). For other high-risk cases (PR ≥ 240 ms and/or QRS > 150–160 ms), a more prolonged hospital stay (3–4 days) should be considered. While awaiting future data, management should be individualized by intra-procedural details (e.g., pre- and post-dilatation, MS length, THV type, oversizing, and implantation depth) along with post-procedural tools such EPS or AECG monitoring.

## 8. Limitations

This review aims to provide an updated overview of the current evidence on new-onset LBBB after TAVI and to summarize the most relevant available literature. However, several limitations should be acknowledged. First, as a narrative review, it does not follow a formal systematic methodology for study selection and may therefore be subject to selection bias. Second, the available evidence is heterogeneous, with variability in study design, patient populations, definitions, and valve types (some now discontinued), limiting direct comparisons across studies.

## 9. Conclusions

The occurrence of new-onset LBBB remains a significant concern in the field of TAVI, as it is associated with an increased risk of PPI, heart failure hospitalization, and mortality. In this context, future efforts should focus on addressing modifiable risk factors to reduce the incidence of LBBB following TAVI.

In the post-procedural period and while awaiting new data, individualized decision-making will be necessary for this group of patients. For those with a stable ECG, normal PR interval, and QRS < 150 ms, discharge at day 2 after the procedure without AECG monitoring appears appropriate. For other cases at higher risk (PR ≥ 190 ms and/or QRS > 150–160 ms), a more prolonged hospital stay (3–4 days) following TAVI may be considered. Factors such as intra-procedural details (e.g., pre- and post-dilatation, MS length, THV type, prosthesis grade of oversizing, implantation depth), day-by-day ECG evolution, and post-procedural clinical tools (EPS, AECG monitoring) should guide the clinical management of these patients.

## Figures and Tables

**Table 1:** Ongoing studies focusing on conduction disturbances after TAVI.

Study	NCT Number	Population	N	Design	Intervention	End Points
TAVI-REVERSE	NCT06481137	Patients undergoing TAVI with an indication for EP study	209	Prospective, observational	EP-guided strategy with pacemaker implantation if HV > 70 ms vs AECG monitoring	Incidence of retrogradation of infra-Hisian CDs
EAGLE-TAVR	NCT06762145	Patients undergoing TAVI without prior PPI	200	Randomized, prospective	Early EP-guided management vs standard care	High-grade CDs and PPI
COME-TAVI	NCT03303612	Patients undergoing TAVI with new-onset LBBB	250	Randomized, prospective	EP-guided strategy vs clinical follow-up with implantable cardiac monitoring	Composite of cardiovascular hospitalization, syncope, or death and incidence of HAVB at 1 year
IMPACT	NCT05308888	Patients undergoing TAVI without prior PPI	100	Prospective, observational	Assessment of local myocardial inflammation by PET	New-onset CDs
AI-Enabled ECG Diagnostic Solution (ZBPro)	NCT06013917	Patients undergoing TAVI with AECG monitoring	75	Prospective feasibility study	Cloud-based artificial intelligence–enabled ECG diagnostic platform	Feasibility and diagnostic accuracy for detection of CDs
PACE-TAVI	NCT05278585	Patients undergoing TAVI without prior PPI	500	Prospective, observational	RAP during the TAVI procedure	PPI

AECG: Ambulatory electrocardiogram; CDs: Conduction disturbances; EP study: electrophysiological study; HAVB: high-degree atrioventricular block; LBBB: left bundle branch block; PET: Positron Emission Tomography; PPI: permanent pacemaker implantation. RAP: Rapid Atrial Pacing.

## Data Availability

No new data were created or analyzed in this study. Data sharing is not applicable to this article.

## Abbreviations

AS	Aortic Stenosis
CDs	Conduction disturbances
CHB	Complete heart block
ECG	Electrocardiogram
EPS	Electrophysiology study
HAVB	High-grade Atrioventricular Block
HV	His-ventricular
LBBB	Left Bundle Branch Block
LVEF	Left ventricular ejection fraction
MS	Membranous Septum
PPI	Permanent Pacemaker Implantation
SAVR	Surgical aortic valve replacement
TAVI	Transcatheter Aortic Valve Implantation
THV	Transcatheter Heart Valve
